# Eco-Anxiety and Mental Health: Correlates of Climate Change Distress

**DOI:** 10.3390/ijerph22121768

**Published:** 2025-11-21

**Authors:** Alessio Mosca, Debora Luciani, Stefania Chiappini, Andrea Miuli, PsyClimate Research Group, Paolo Cianconi, Mauro Pettorruso, Luigi Janiri, Giovanni Martinotti

**Affiliations:** 1 Department of Neuroscience, Imaging and Clinical Sciences, “G. d’Annunzio” University of Chieti-Pescara, 66100 Chieti, Italymauro.pettorruso@hotmail.it (M.P.);; 2 Department of Human and Clinical Sciences, UniCamillus—International Medical University, Via di Sant’Alessandro 8, 00131 Rome, Italy; 3 Psychopharmacology, Drug Misuse and Novel Psychoactive Substances Research Unit, School of Health, Medicine and Life Sciences, University of Hertfordshire, Hatfield AL10 9EU, UK; 4 Department of Human Science, Libera Università Maria SS. Assunta, 00193 Rome, Italy; 5 Department of Neuroscience, Catholic University of the Sacred Heart, 00168 Rome, Italy

**Keywords:** eco-anxiety, climate change, mental health, psychological distress, psychopathology

## Abstract

Background. Climate change is increasingly recognized as a threat to mental health, giving rise to constructs such as eco-anxiety and solastalgia. Although these phenomena have gained attention, quantitative data from European populations remain scarce. Objectives. This study investigated the prevalence and correlates of eco-anxiety in an Italian sample, focusing on associations with demographics, environmental disaster exposure, psychological distress, psychosis-risk indicators, and quality of life. Methods. A cross-sectional online survey was conducted with 1051 participants. Measures included the Hogg Eco-Anxiety Scale (HEAS-13), Brief Symptom Inventory (BSI), Prodromal Questionnaire (PQ-16), SF-36 Health Survey, and a socio-demographic/environmental exposure questionnaire. Data were analyzed using correlations, t-tests, and regression analyses. Results. Eco-anxiety was higher among younger participants, women, and individuals without children, while education showed a weak negative association. No differences emerged by rural vs. urban residence. Participants exposed to floods, droughts, wildfires, and landslides reported elevated eco-anxiety. Scores correlated strongly with general distress (r = 0.57), positively with psychosis-risk indicators (PQ-16 distress, r = 0.42), and negatively with quality of life (r = −0.25). Conclusions. Eco-anxiety in Italy is linked to socio-demographic vulnerabilities, disaster exposure, and impaired mental health. These findings highlight eco-anxiety as a pressing public health concern and stress the need for prevention and interventions that promote adaptive coping and engagement with climate change.

## 1. Introduction

Climate change constitutes one of the most pressing global health challenges of the 21st century, with far-reaching consequences that extend beyond environmental degradation to deeply affect psychological and social well-being [1,2]. These impacts are generating growing concern and adversely affecting both physical and mental health, to the extent that the World Health Organization (WHO) recognizes it as an unparalleled priority [3]. Rising temperatures, loss of biodiversity, and the increasing frequency of extreme weather events (e.g., floods, wildfires, droughts) have both direct and indirect negative effects on mental health, including increased rates of anxiety, depression, post-traumatic stress disorder (PTSD), and other psychological disturbances [4,5,6].

In recent years, researchers have introduced novel constructs such as Eco-anxiety and Solastalgia to better capture the complex emotional responses elicited by environmental change. Eco-anxiety refers to the chronic fear and worry about the future resulting from awareness of climate change and ecological degradation [7]. Adolescents and young people, in particular, appear to be especially vulnerable: studies report that a large proportion of youth experience turmoil related to climate pressures, including grief, fear, anger, and depression [8].

On the other hand, solastalgia, a term coined by Albrecht (2007) [9], describes the emotional distress experienced when one’s home environment undergoes unwelcome and often irreversible transformation. Unlike nostalgia -longing for a distant past- Solastalgia reflects a sense of loss and unease while still present in one’s environment [9]. Recent reviews indicate that solastalgia is significantly associated with mental health issues such as depression, anxiety, somatic symptoms, and PTSD, particularly in contexts of ongoing environmental destruction [9,10].

From a psychological perspective, climate change has increasingly been recognized not only as an environmental and social issue but also as a profound psychological one. Pioneering work by Susan Clayton (2020) and colleagues [9,11] has conceptualized climate anxiety as a multidimensional emotional response encompassing cognitive, affective, and behavioral components, ranging from adaptive worry to clinically significant distress. According to Clayton, such responses are shaped by individual differences, social context, and coping resources, and should not be pathologized but understood within the broader framework of environmental psychology and public mental health. Similarly, the American Psychological Association (APA) reports [9,11,12] have emphasized the role of psychology in identifying both the risks and the adaptive potential of climate-related emotions, highlighting mechanisms such as self-efficacy, meaning-focused coping, and social connectedness. In line with this theoretical grounding, the present study adopts a psychological approach focused on eco-anxiety, defined as persistent worry and emotional disturbance related to climate change, and measured through the Hogg Eco-Anxiety Scale. This focus distinguishes eco-anxiety from broader constructs such as climate change anxiety, while also acknowledging the related but distinct notion of solastalgia, a place-based form of environmental distress discussed conceptually but not empirically measured in the present study.

Empirical evidence increasingly recognizes that climate-related psychological distress does not affect all populations equally. Vulnerable groups, including children, elderly individuals, those with pre-existing mental health conditions, and people of lower socioeconomic status, are disproportionately impacted by eco-distress and solastalgia [6].

Furthermore, systematic reviews highlight how eco-anxiety tends to co-occur with elevated psychological distress, while solastalgia exacerbates mental health harms through feelings of helplessness and loss of solace [13].

At the European level, an increasing number of studies have begun to quantify the mental-health consequences of climate-related concern. Large cross-national surveys by [14,15] demonstrated that perceived climate threat and environmental worry are consistently associated with psychological distress, reduced well-being, and pro-environmental engagement across several European populations. These contributions highlight that Europe, while no longer devoid of data, still lacks comprehensive investigations integrating validated psychometric instruments and standardized clinical indicators of mental health. Within this context, the present research aims to extend the European evidence base by providing a detailed quantitative analysis of eco-anxiety and its psychological correlates. In recent years, a number of European and Italian studies have begun to investigate the psychological dimensions of climate change and their implications for mental health. At the European level several studies have documented how climate-related concern and exposure predict heightened psychological distress and reduced well-being [14,15]. Within the Italian context, emerging research has examined eco-anxiety and related constructs such as environmental worry and solastalgia, highlighting associations with depressive and anxiety symptoms, emotional regulation, and pro-environmental behavior [16,17,18]. By building on this growing body of work, the present study extends previous research by providing a comprehensive quantitative analysis of eco-anxiety and its correlates in a large Italian sample, thereby contributing to the development of a cross-cultural understanding of climate-related psychological distress.

Recent research has also highlighted the link between climate-related distress and reproductive decision-making. Climate anxiety and perceptions of environmental threat have been shown to shape fertility intentions and parental concerns, especially among younger adults who express uncertainty about bringing children into a climate-threatened world [11,19,20]. Including parental status as a demographic variable therefore provides insight into possible intergenerational and existential dimensions of eco-anxiety.

Despite growing scholarly interest, quantitative research remains limited, especially in European contexts. Many existing studies offer descriptive or qualitative insights, while few systematically explore the interrelationships between eco-anxiety, solastalgia, demographic variables, general mental health indicators, and quality of life metrics. Addressing this gap has significant implications for developing targeted prevention and intervention strategies that integrate mental health support within climate response frameworks.

### Study’s Objectives

The present study aims to address these lacunae by investigating eco-anxiety and its psychological correlates in an Italian sample. By embedding eco-distress within a robust empirical framework, this research contributes to the emerging field of climate-psychological health and informs culturally appropriate interventions. Using validated psychometric instruments (e.g., HEAS, BSI, PQ-16, SF-36), the study examines:

(1) The prevalence and demographic correlations of eco-anxiety;
(2) Its association with general psychological distress and psychosis-risk indicators;
(3) Its relationship with perceived quality of life.

## 2. Materials and Methods

### 2.1. Participants

The study employed a cross-sectional, survey-based design. Participants were recruited through online advertisements (e.g., social media, mailing lists) and completed the survey anonymously via Google Forms. Eligibility criteria included being at least 18 years old and providing informed consent. The final sample consisted of 1051 participants aged 15 to 89 years (M = 34.25, SD = 15.0). The majority identified as female (68.9%), followed by male (30.6%) and other gender identities (0.5%). Most participants reported upper secondary or higher education (84%), and approximately 69% had no children. Participants were recruited from diverse regions of Italy through online platforms and mailing lists, ensuring broad geographic coverage. Detailed demographic characteristics are presented in Table 1.

The online survey required approximately 20 min to complete. It consisted of two sections: (a) socio-demographic and environmental exposure questions, and (b) the psychometric instruments (HEAS-13, BSI, PQ-16, SF-36). Data were exported into an Excel database and analyzed with IBM SPSS Statistics version 25. Participation was voluntary and anonymous. All participants provided written informed consent prior to participation. The study protocol was approved by the internal ethics committee of the University and was conducted in accordance with the principles outlined in the Declaration of Helsinki.

### 2.2. Measures

- Socio-demographic and environmental exposure questionnaire: participants completed a bespoke questionnaire collecting socio-demographic characteristics, medical and psychiatric history, and multi-domain indicators of environmental exposure and climate-related perceptions (Google Forms; items listed below). Unless otherwise specified, higher numeric values indicate greater intensity of the construct. Item wording and response options derive from the original Italian survey form.
  - Socio-demographics. Age (in years), gender (female/male/prefer not to say), occupational status (student/professional/employee/unemployed/retired/other), educational attainment (six-level ordinal scale: primary school to master’s/PhD), and parental status (no children/one/more than one). Municipality size was reported in population brackets: <10,000; 10,000–50,000; 50,000–250,000; 250,000–1,000,000; >1,000,000.
  - Health history. Current chronic physical illness (yes/no) and related medications (free text); current psychiatric disorder (yes/no) and psychotropic medications (free text); family history of psychiatric disorders in first-/second-degree relatives (yes/no).
  - Residential context and rootedness. Rural context (multi-select: lives in city; near sea; near mountains; near countryside; other). Length and depth of local rootedness were captured with a graded item (e.g., “a few years”, “same municipality as birth”, extending to “same municipality for generations”).
  - Climate-related mobility and work. Lifetime climate-related relocation (yes/no) and climate-related job change (yes/no). Perceived impact of climate change on one’s job was rated on a 0–10 scale (“not at all” to “completely”).
  - Exposure to natural hazards. Lifetime exposure (multi-select) to: floods; drought/desertification; wildfires; hurricanes/tornadoes; landslides/avalanches; coastal erosion; tsunami; none. Post-disaster suicidality was assessed with a single yes/no item (“following a natural disaster, have you ever thought about or attempted to take your life?”).
  - Local environmental change and climate perceptions. Participants rated: (a) extent to which the local environment has changed due to climate change or natural disasters (0–10); (b) perceived worsening of air pollution in the current place of residence (0–10); (c) perceived change in local temperatures (rise/fall/no change); and (d) personal priority given to climate change (0–10; “not at all” to “extremely”).
  - Self-rated climate impact on health (key variables). Two items captured perceived climate-related impacts on health: (a) “To your knowledge, has climate change affected your physical health?” (0–10); (b) “To your knowledge, has climate change affected your mental health?” (binary yes/no), followed—if yes—by “To what extent?” rated 0–10.
- Hogg Eco-Anxiety Scale (HEAS-13) [21]: Eco-anxiety was assessed with the 13-item HEAS, comprising four subscales: Affective Symptoms (4 items), Rumination (3 items), Behavioral Symptoms (3 items), and Personal Impact (3 items). Items are rated on a 0–3 scale, and subscale scores were computed as item means. Consistent with the theoretical framework of the scale, higher scores reflect a greater frequency of climate-related emotional, cognitive, and behavioral experiences.
- Brief Symptom Inventory (BSI) [22]: Psychological distress was measured using the BSI, a 53-item self-report instrument that assesses nine symptom dimensions (e.g., depression, anxiety, somatization, hostility). Three global indices were also calculated: Global Severity Index (GSI), Positive Symptom Distress Index (PSDI), and Positive Symptom Total (PST).
- Prodromal Questionnaire—16 items (PQ-16) [23]: The PQ-16 screened for prodromal psychotic experiences. It consists of 16 true/false items, followed by ratings of distress on a 4-point scale. Both the number of endorsed symptoms and the weighted distress scores were computed.
- SF-36 Health Survey [24]: Health-related quality of life was evaluated with the SF-36, which yields a total score as well as subscale indices of physical and mental functioning.

All psychometric instruments were administered in their validated Italian versions. Specifically, the Italian adaptation of the Hogg Eco-Anxiety Scale (HEAS) was used [17], which demonstrated good internal consistency (Cronbach’s α = 0.89 in the original validation). The scale assesses four dimensions of eco-anxiety—affective symptoms, rumination, behavioral symptoms, and personal impact—on a 4-point Likert scale. Consistent with previous research supporting a higher-order single factor [17,21], the present study primarily analyzed the total HEAS score to represent overall eco-anxiety. Psychological distress was measured with the Italian version of the Brief Symptom Inventory [25] (α = 0.96), while the Prodromal Questionnaire–16 was administered using its Italian validation [26] (α = 0.84). Health-related quality of life was assessed with the Italian SF-36 Health Survey [27] (α = 0.90).

In the present study, internal consistency indices were acceptable to excellent across measures (Cronbach’s α ranging from 0.78 to 0.93; McDonald’s Ω from 0.80 to 0.94).

### 2.3. Statistical Analyses

Descriptive statistics (mean, standard deviation, minimum, and maximum) were computed for all variables. Distributional properties were examined by calculating unbiased sample skewness (g_1_) and excess kurtosis (g_2_), as well as conducting Shapiro–Wilk tests of univariate normality. Following standard psychometric guidelines, absolute skewness values ≤ 2 and kurtosis values ≤ 7 were considered acceptable for parametric analyses in large samples.

Prior to correlation and regression analyses, all statistical assumptions were verified. Linearity among continuous variables was confirmed through visual inspection of scatterplots and standardized residuals. Multicollinearity was assessed by computing Variance Inflation Factor (VIF) and Tolerance values, which indicated no collinearity problems (all VIF < 2.5; Tolerance > 0.40). The presence of outliers or influential observations was evaluated through standardized residuals, leverage, and Cook’s distance. No cases exceeded conventional thresholds (Cook’s D < 1.0; leverage < 0.02), confirming that the data met the assumptions for parametric analyses.

Descriptive statistics (means, standard deviations, and frequencies) were computed for all variables. Pearson correlations were conducted between HEAS scores and continuous variables (e.g., age, education, BSI, PQ-16, SF-36). Group differences in HEAS scores (e.g., gender, parental status, disaster exposure) were examined using independent-samples t tests and one-way ANOVAs. Linear regression analyses were then performed to assess the predictive associations between eco-anxiety (independent variable) and psychological distress and quality of life (dependent variables). Statistical significance was set at *p* < 0.05 (two-tailed).

Coding and data handling. Ordinal and Likert-type items were analyzed as continuous variables when distributions were approximately symmetric; binary items were coded 0/1. Multi-select hazard exposures (e.g., floods, wildfires) were recoded into separate dummy variables (0 = no, 1 = yes). Free-text medication fields were retained for descriptive purposes only and excluded from inferential analyses. Item-level missingness was < 5%; correlations were estimated using pairwise deletion, whereas regression models used listwise deletion. Sensitivity analyses with multiple imputations (five imputations, fully conditional specification) yielded comparable results and are reported in the Supplementary Materials.

Planned uses in analysis. In addition to descriptive statistics, selected variables were entered as predictors or covariates in correlation and regression models. These included municipality size, rural context, rootedness, climate-related relocation or job change, hazard exposures, indices of local environmental change (environmental degradation, air pollution, temperature change), climate priority, and the three self-rated climate–health items (physical health, mental health yes/no, and extent of mental health impact). These were examined for their associations with eco-anxiety and mental health outcomes (BSI, PQ-16, SF-36) alongside the HEAS.

Pearson correlation coefficients were computed between each HEAS subscale (Affective, Rumination, Behavioral, Personal Impact) and the main psychological measures (BSI, PQ-16, SF-36) to examine the pattern of associations across domains. Correlation values were summarized in a dedicated table.

## 3. Results

The final sample consisted of 1051 participants (68.9% female, 30.6% male, 0.5% other; M age = 34.25, SD = 15.00, range = 15–89). Demographic characteristics of the sample are presented in Table 1. Preliminary checks confirmed that the distributions of the main study variables met the assumptions for parametric analysis. As expected for psychological measures, the HEAS, BSI, and PQ-16 scores showed moderate positive skewness (|g_1_| < 1.4; |g_2_| < 2), while the SF-36 displayed a marked negative skew (g_1_ = −3.12) and high kurtosis (g_2_ = 15.47), reflecting a ceiling effect common in well-being indices. According to the established thresholds (|g_1_| ≤ 2; |g_2_| ≤ 7), all scales except the SF-36 showed acceptable departures from normality. Shapiro–Wilk tests were significant for most variables due to the large sample size (N = 1051), but visual inspection and skewness/kurtosis indices indicated distributions suitable for parametric tests. A comprehensive table reporting descriptive statistics for each variable (mean, SD, min, max, skewness, kurtosis, and Shapiro–Wilk W and *p* values) has been included in the Supplementary Materials (Table S1).

### 3.1. Eco-Anxiety and Demographic Variables

Pearson correlations revealed a significant negative association between eco-anxiety (HEAS total score) and age (r = −0.19, *p* < 0.001), indicating higher levels of eco-anxiety among younger participants (i.e., individuals under 35 years, corresponding to the lowest tertile of the age distribution). Eco-anxiety was also negatively associated with number of children (r = −0.18, *p* < 0.001), with childless participants reporting greater levels of eco-anxiety. Regarding education, a small but significant negative correlation emerged (r = −0.11, *p* < 0.001), suggesting that individuals with lower educational attainment reported slightly higher eco-anxiety.

Independent-samples t-tests showed significant gender differences, with women scoring higher on the HEAS compared to men (M = 3.32 vs. M = 2.34; t(1049) = 4.41, *p* < 0.001). No significant differences were found for rural versus non-rural residence (t(1049) = 0.47, *p* = 0.64) or size of place of residence (r = −0.01, *p* = 0.75) (see Table 2).

### 3.2. Eco-Anxiety and Psychological Outcomes

Eco-anxiety was strongly correlated with general psychological distress as measured by the Brief Symptom Inventory (BSI; r = 0.57, *p* < 0.001). A positive correlation also emerged with psychosis-risk indicators on the Prodromal Questionnaire (PQ-16), both for the number of endorsed symptoms (r = 0.11, *p* < 0.001) and for distress-weighted scores (r = 0.42, *p* < 0.001). Finally, eco-anxiety was negatively associated with health-related quality of life (SF-36 total score; r = −0.25, *p* < 0.001), indicating that higher levels of eco-anxiety were linked to lower perceived well-being (see Table 2). All HEAS subscales showed significant associations with psychological distress (BSI) and psychotic-like experiences (PQ-16), with the strongest effects observed for the Affective Symptoms subscale, followed by Behavioral Symptoms and Personal Impact. Rumination showed weaker correlations. All associations with SF-36 were negative, indicating lower perceived quality of life. Detailed correlation coefficients are reported in Table 3.

### 3.3. Eco-Anxiety and Environmental Exposure

Eco-anxiety scores were significantly higher among participants who had experienced floods (t(928) = 2.46, *p* = 0.014), droughts (t(1049) = 4.22, *p* < 0.001), wildfires (t(1049) = 4.40, *p* < 0.001), and landslides/avalanches (t(1049) = 4.85, *p* < 0.001). No significant difference was found for exposure to hurricanes/tornadoes (t(1049) = 0.58, *p* = 0.56). Group comparisons of HEAS scores across categorical variables are reported in Table 4. Corresponding effect sizes (Cohen’s d) indicated small-to-moderate magnitude differences for gender (d = 0.30) and environmental exposures such as floods, droughts, wildfires, and landslides (d range = 0.22–0.39). These values suggest that, while statistically significant, group differences in eco-anxiety were of modest practical size, consistent with previous population-based findings on climate-related emotions.

### 3.4. Perceived Climate-Related Impacts on Health

A substantial proportion of participants reported that climate change had negatively affected their health. On a 0–10 scale, the mean self-reported impact on physical health was M = 2.77 (SD = 2.74), whereas mental health impact was endorsed by 63.6% of the sample (yes vs. no). Among those endorsing mental health impact, the average severity rating was M = 2.88 (SD = 2.95).

Correlation analyses revealed that these subjective perceptions were significantly associated with psychometric indices (Table 5). Perceived impact on mental health (0–10 scale) correlated positively with eco-anxiety (r = 0.41, *p* < 0.001), general psychological distress (BSI; r = 0.32, *p* < 0.001), and psychosis-risk indicators (PQ-16; r = 0.26, *p* < 0.001). Similar but slightly weaker associations were observed for perceived impact on physical health (eco-anxiety: r = 0.31; BSI: r = 0.23; PQ-16: r = 0.22, all *p* < 0.001).

**Figure 1:**
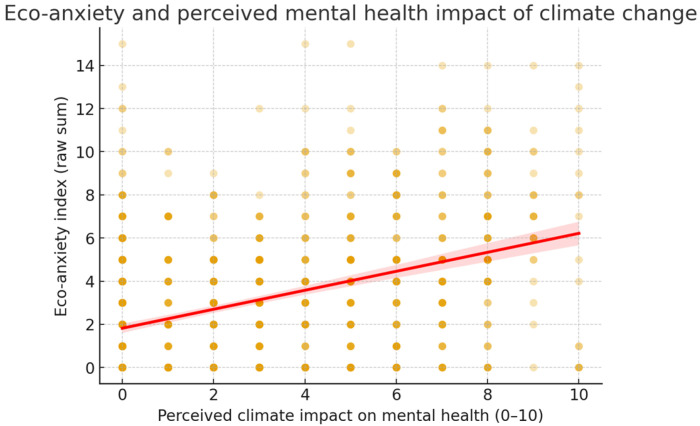
Association between perceived climate impact on mental health and eco-anxiety. Scatterplot with regression line showing that higher self-rated impact (0–10) corresponds to higher eco-anxiety scores.

**Figure 2:**
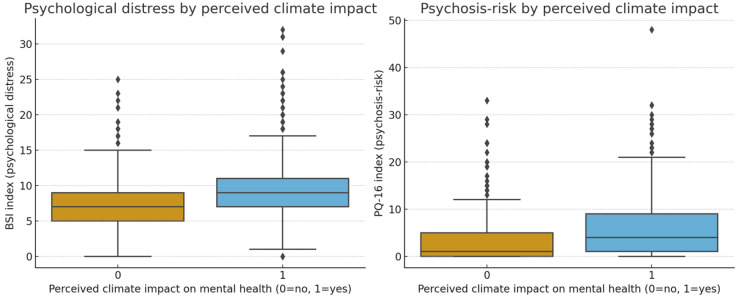
Boxplots of BSI (psychological distress) and PQ-16 (psychosis-risk) scores by endorsement of perceived climate-related mental health impact (yes vs. no). Participants endorsing impact consistently reported higher distress and risk indicators.

The scatterplot further illustrates the linear association between self-rated mental health impact and eco-anxiety levels (Figure 1). Group comparisons confirmed that participants who reported that climate change had affected their mental health (yes vs. no) scored significantly higher on eco-anxiety, BSI, and PQ-16 indices (all *p* < 0.001; see Figure 2).

These findings indicate that individuals’ subjective appraisal of climate change as detrimental to their own health is closely linked with elevated eco-anxiety and broader mental health vulnerabilities.

## 4. Discussion

The present study investigated the associations between eco-anxiety, socio-demographic characteristics, environmental exposures, and mental health outcomes in a large Italian sample. Several important findings emerged that contribute to the growing body of literature on the psychological consequences of climate change.

### 4.1. Eco-Anxiety and Socio-Demographic Factors

Consistent with prior work, younger age was strongly associated with higher levels of eco-anxiety [11,29,30]. Several mechanisms may account for this pattern. Adolescence and early adulthood are periods of ongoing psychosocial and neurobiological maturation, characterized by identity consolidation and heightened affective reactivity [31], which can increase sensitivity to climate-related stressors. Younger cohorts are also disproportionately exposed to climate content through social and digital media, where coverage is frequent, often alarm-framed, and not always scientifically vetted; such exposure can amplify perceived threat and uncertainty about the future. Another plausible explanation is that adolescents and young adults are the cohorts most sensitive to postmodern societal transformation. Over the past two decades, rapid and often dramatic social changes have altered patterns of individualization, social roles, identity and intimacy, and people’s relationship to temporality [31,32,33]. These shifts have been accelerated by the widespread adoption of the internet and new technologies, which intensify interconnectivity, digitalization, and platform-mediated entertainment, contributing to a pervasive sense of time pressure and a “culture of urgency” [34]. Exposure to these changes has increased youths’ vulnerability and risk for psychological distress [31].

Finally, time-horizon effects likely contribute: compared with older adults, young people expect to live longer with the consequences of climate change and therefore perceive personal risk as more immediate and enduring. Together, these factors plausibly elevate eco-anxiety among youth and help explain their greater engagement with environmental issues.

Women reported significantly higher levels of eco-anxiety than men, consistent with prior evidence of greater emotional reactivity and vulnerability to climate-related stressors among females [35]. Several sociocultural and psychological mechanisms may contribute. Caregiving and household roles can heighten vigilance to environmental threats perceived as jeopardizing domestic well-being. In addition, women show higher baseline rates of anxiety [36] and a stronger prosocial/communal orientation, including greater social responsibility, which correlate with environmental concern and sustainable behaviors [37]. These factors may account for the observed -albeit small but significant- association between female gender and elevated eco-anxiety. Another plausible mechanism is that greater involvement in childrearing may heighten future-oriented empathy, concern for children’s and grandchildren’s exposure to climate risks, thereby intensifying eco-anxiety. However, this stands in tension with our finding that participants without children reported higher eco-anxiety, plausibly reflecting intensified concerns about reproductive futures and intergenerational justice [38]. Among women, eco-anxiety was inversely related to parity: those with fewer children scored higher, and we observed a small but significant negative correlation between HEAS [21] scores and number of children overall. Several non-exclusive mechanisms may account for these patterns: greater per-child emotional investment and anticipatory worry; more time and cognitive bandwidth to engage with climate information when caring for fewer dependents; and, conversely, for larger families, broader support networks and everyday demands that provide distraction or facilitate coping. A selection effect is also plausible—individuals with pronounced eco-anxiety may defer or avoid childbearing—such that parenthood may function as a modest protective factor for some, while concerns for future generations amplify distress among the childless. This interpretation aligns with recent research indicating that climate-related emotions can influence reproductive attitudes and parental decision-making. Surveys have shown that individuals experiencing strong eco-anxiety or climate-related worry sometimes delay or reconsider parenthood out of concern for their children’s future and the planet’s livability [11,19]. Such findings support viewing eco-anxiety as an emotion that extends beyond individual well-being to encompass intergenerational and ethical dimensions.

Education showed a small but significant inverse association with eco-anxiety, a pattern not consistently reported in prior studies. One interpretation is that higher educational attainment supports more analytic processing and environmental literacy, fostering perceived efficacy, problem-focused coping, and the ability to situate climate risks within broader scientific and policy frameworks. Moreover, highly educated individuals may be embedded in socio-occupational contexts with competing daily demands that reduce the salience of climate threats. Greater access to reliable information and practical resources could also render environmental risks more tractable, attenuating anxiety. This counterintuitive finding merits further investigation, including tests of mediators (e.g., perceived control, risk appraisal, media diet, nature connectedness) and socioeconomic confounders, as well as longitudinal designs to clarify directionality. These findings are consistent with recent European and Italian research that has documented similar associations between climate-related emotions and mental health. Rocchi and colleagues reported higher levels of eco-anxiety among younger and female participants in the Italian population and found that climate-related worry and emotional distress were linked to reduced well-being and a sense of uncertainty about the future. Moreover, Abate and colleagues highlighted how solastalgia and place-based environmental concern are associated with depressive and anxiety symptoms [18]. The present study extends these findings by examining eco-anxiety together with psychosis-risk indicators and quality of life, thereby contributing to the consolidation of an Italian and European evidence base on the mental-health impacts of climate change. Recent literature confirms that eco-anxiety and related emotional responses are particularly salient among young people and women. Emerging adults report higher levels of climate-related worry and distress, reflecting both developmental sensitivity and future-oriented concerns [39,40]. Such emotions, though often distressing, may also foster environmental engagement and collective action [11]. Consistent with our findings, women tend to report stronger eco-anxiety, possibly due to higher empathic concern and socialization patterns emphasizing care and relational responsibility [19,41]. These results underscore how sociocultural and gendered expectations may shape emotional responses to environmental threats.

Moreover, the role of media and social media is increasingly recognized as a key contextual factor. Exposure to climate change information through digital platforms can intensify emotional reactions, contributing to feelings of helplessness and threat [42], yet social media also functions as a channel for collective coping and activism, promoting agency and social connectedness [11]. This ambivalence highlights the need to balance informative and efficacy-oriented climate communication, especially among younger audiences, to prevent maladaptive anxiety while fostering engagement.

Taken together, these findings suggest that interventions and educational programs addressing eco-anxiety should be age- and gender-sensitive, emphasizing empowerment, community dialog, and constructive engagement through trusted media ecosystems.

### 4.2. Environmental Exposures

Exposure to specific natural disasters -floods, droughts, wildfires, and landslides- was significantly associated with higher eco-anxiety, whereas exposure to hurricanes/tornadoes was not. A parsimonious explanation for this pattern is that personally experiencing a catastrophic natural event, or even perceiving slow environmental degradation, heightens distress related to climate change [43]. Our findings extend this view by showing that slow-onset hazards, such as droughts, are likewise linked to increased eco-anxiety. This underscores that eco-anxiety is not merely a reaction to acute, dramatic events; it can also arise from chronic environmental stressors that erode feelings of stability and security in daily life [9].

### 4.3. Eco-Anxiety and Mental Health Outcomes

As expected, eco-anxiety showed a robust correlation with psychological distress, consistent with its overlap with anxiety, depressive, and stress-related symptomatology [6,35]. This finding supports an association between eco-anxiety and psychopathology; however, the directionality of this relationship remains unclear and warrants further investigation. We hypothesize that, because Italy is not frequently exposed to extreme events or abrupt climate shifts, elevated eco-anxiety may, at least in part, reflect underlying levels of psychopathology. Accordingly, individuals with pre-existing mental disorders, particularly mood and anxiety disorders, may be more vulnerable to climate-change-related distress.

Furthermore, eco-anxiety was positively associated with psychotic-like experiences on the PQ-16, particularly when distress weighting was considered. As with any cross-sectional association, the direction of effect is uncertain: eco-anxiety may heighten vulnerability to psychotic psychopathology, or, conversely, psychotic liability may amplify eco-anxious appraisals. In the Italian context -where recent climate impacts have been comparatively moderate- the latter hypothesis is plausible, namely that psychotic symptomatology contributes to elevated eco-anxiety. Clinically, individuals with psychotic disorders frequently report pronounced fears of annihilation at both the personal and collective level [44].

Phenomenological psychiatry has long characterized an ‘end-of-the-world’ or apocalyptic atmosphere in emerging psychosis: Conrad’s Trema phase and apophany, Jaspers’s delusional mood, Minkowski’s disturbances of lived time, and Blankenburg’s loss of natural self-evidence all describe a collapse of the taken-for-granted world and a sense of impending catastrophe [45,46,47,48], themes that also resonate with contemporary psychopathology [49]. From this perspective, climate change may be interpreted within a preexisting catastrophic experiential frame, rendering apocalyptic meanings more salient and thereby intensifying eco-anxiety. Relatedly, De Martino’s analyses of cultural “end-of-the-world” scenarios and the crisis of presence underscore how collective apocalyptic narratives can interact with individual vulnerability to produce experiences of existential threat [50].

In line with earlier evidence, eco-anxiety was negatively correlated with health-related quality of life (SF-36), indicating that elevated eco-anxiety is linked to impaired well-being and functioning [27].

The subscale results indicate that the affective component of eco-anxiety is most closely associated with psychological distress and functional outcomes. Behavioral interference and perceived personal impact provided smaller but meaningful contributions, while rumination showed limited independent effects after accounting for the other components. These findings support the multidimensional structure of the HEAS and clarify the relative contribution of its components.

### 4.4. Perceived Climate-Related Health Impacts and Psychological Outcomes

In addition to standardized psychometric scales, our study incorporated self-rated items assessing the perceived impact of climate change on physical and mental health. These variables proved highly informative: nearly two-thirds of the sample endorsed some impact on mental health, and severity ratings correlated robustly with eco-anxiety, general psychological distress, and psychosis-risk indicators. Participants perceiving a climate-related deterioration in their health consistently scored higher on eco-anxiety, BSI, and PQ-16 indices.

These findings highlight that eco-anxiety is not experienced in isolation from individuals’ broader health perceptions. Rather, subjective appraisal of climate-related harm appears to function as an experiential bridge linking environmental concern with psychological distress. This is consistent with literature showing that self-rated health is a strong predictor of mental health outcomes and that perceived vulnerability to climate change exacerbates anxiety and depressive symptoms [20,43,51].

Clinically, these results underscore the importance of assessing not only abstract climate concerns but also how individuals experience climate change as impacting their own health. Such perceptions may represent both a marker of vulnerability and a potential target for intervention. Addressing maladaptive appraisals and fostering adaptive coping strategies could mitigate distress while validating legitimate concerns about environmental degradation.

### 4.5. Limitations and Future Directions

Several limitations should be noted. First, the cross-sectional design prevents causal inferences about the direction of relationships between eco-anxiety and mental health outcomes. Second, all data were self-reported, raising the possibility of reporting biases. Third, the sample, though large, may not be representative of the Italian population, as it was recruited online and skewed toward younger and more educated participants.

Future research should adopt longitudinal and cross-cultural designs, include more diverse samples, and investigate protective factors such as social support, resilience, and pro-environmental behaviors that may buffer the negative impacts of eco-anxiety. Such studies would help clarify causal pathways, identify protective factors, and test targeted interventions. Special attention should be paid to vulnerable groups such as youth, women, individuals without children, and those with pre-existing mental health conditions, as well as to the role of subjective perceptions of health impacts. By bridging empirical findings with clinical and policy strategies, addressing eco-anxiety and solastalgia can become a cornerstone in building climate-resilient mental health systems.

## 5. Conclusions

Recognizing eco-anxiety as both a public mental health concern and a potential motivator for adaptive engagement with climate change is crucial for clinical practice and public policy. The present study, based on a large Italian sample, contributes novel evidence by systematically examining eco-anxiety alongside demographic vulnerabilities, exposure to environmental hazards, general psychological distress, psychosis-risk indicators, quality of life, and, importantly, self-rated perceptions of climate-related health impacts. These findings underscore that eco-anxiety is not an isolated construct but is embedded within broader patterns of vulnerability, lived experience, and subjective health appraisals.

From a public health perspective, the results call for integrating climate-related psychological distress into prevention and intervention frameworks. This may include psychoeducation, clinical screening, and therapeutic approaches (e.g., cognitive-behavioral therapy, ecotherapy, mindfulness-based interventions) that help individuals regulate distress while validating legitimate environmental concerns [52]. At the societal level, strengthening resilience, social support, and collective efficacy may buffer the psychological costs of climate change and facilitate adaptive engagement.

From an applied perspective, these findings underscore the need for integrating climate-related psychological distress into public health and clinical frameworks. Mental health professionals, psychologists, psychiatrists, and psychotherapists should be trained to recognize and address eco-anxiety within diagnostic and therapeutic contexts, using approaches such as cognitive-behavioral therapy, ecotherapy, and mindfulness-based interventions. Educators and youth organizations can play a preventive role by promoting emotional awareness, resilience, and constructive engagement with environmental issues among younger generations. Policy makers and public health agencies should incorporate psychological well-being into national climate adaptation and mitigation strategies, ensuring that mental health is recognized as a key dimension of climate resilience. Finally, closer collaboration between researchers in psychology, psychiatry, environmental science, and public health is needed to develop evidence-based, cross-sectoral interventions that support individuals and communities in facing the mental health challenges of climate change.

## Figures and Tables

**Table 1:** Demographic characteristics of the sample.

Characteristic	Value
N	1051
Age (M ± SD)	34.25 ± 15.01
Age range	15–89
Gender	Female: 724 (68.9%); Male: 322 (30.6%); Other: 5 (0.5%)
Education	Level 1: 2 (0.2%); Level 2: 70 (6.7%); Level 3: 440 (41.9%); Level 4: 266 (25.3%); Level 5: 180 (17.1%); Level 6: 93 (8.8%)
Children	No children: 727 (69.2%); One child: 105 (10.0%); Two or more: 219 (20.8%)

Note. Age is reported as mean ± standard deviation (SD) and range. Education was coded on a 6-point ordinal scale: 1 = primary school, 2 = lower secondary school, 3 = upper secondary school, 4 = post-secondary non-tertiary, 5 = bachelor’s degree, 6 = master’s degree or higher. Percentages may not total 100% due to rounding.

**Table 2:** Correlations between eco-anxiety (HEAS) and continuous variables.

Variable	r	*p*	95% CI
Age	−0.185	<0.001	(−0.243, −0.126)
Education	−0.107	<0.001	(−0.166, −0.046)
Children	−0.176	<0.001	(−0.234, −0.117)
BSI_total	0.567	<0.001	(0.524, 0.607)
PQ_total	0.114	<0.001	(0.054, 0.173)
PQ_distress	0.421	<0.001	(0.37, 0.47)
SF36_total	−0.252	<0.001	(0.194, 0.308)

Note. Pearson correlation coefficients (r) are reported with *p*-values and 95% confidence intervals (CI). Negative coefficients indicate higher eco-anxiety is associated with lower values on the variable (e.g., age, education, children, quality of life). Positive coefficients indicate direct associations (e.g., psychological distress). All correlations were significant at *p* < 0.001.

**Table 3:** Correlations between HEAS subscales and psychological variables.

Outcome Variable	Affective	Rumination	Behavioral	Personal Impact
BSI Total	0.585	0.265	0.529	0.399
PQ-16 Total	0.531	0.321	0.487	0.409
SF-36 Total	−0.109	−0.120	−0.115	−0.030

Note. Values are Pearson’s r. Positive correlations indicate higher psychological distress or psychotic-like experiences; negative correlations indicate poorer quality of life.

**Table 4:** Group comparisons of eco-anxiety (HEAS total score).

Comparison	Female Mean	Male Mean	t	df	*p*	Cohen_d	95% CI d	Yes_Mean	No_Mean
Gender (Female vs. Male)	3.32	2.34	4.41	1044	<0.001	295	(0.163, 0.427)		
Floods			2.46	928	0.01426	221	(0.044, 0.398)	3.68	2.93
Droughts			4.22	1049	<0.001	339	(0.181, 0.497)	3.96	2.84
Wildfires			4.4	1049	<0.001	0.28	(0.155, 0.405)	3.61	2.68
Landslides			4.85	1049	<0.001	0.39	(0.232, 0.549)	4.1	2.81
Hurricanes			0.58	1049	0.55985	78	(−0.183, 0.338)	3.28	3.02

Note. Independent-samples *t*-tests were used to compare HEAS scores across groups. Group means (M), t-values, degrees of freedom (df), *p*-values, Cohen’s d (effect size), and 95% confidence intervals (CI) for d are reported. Small effects correspond approximately to d = 0.20, medium effects to d = 0.50, and large effects to d = 0.80 [28]. All correlations were significant at *p* < 0.001.

**Table 5:** Descriptive statistics and correlations of perceived climate-health impact variables with eco-anxiety and psychological outcomes.

Variable	Mean	SD	r with EcoAnxiety Index	r with BSI Index	r with PQ16 Index
Perceived climate impact on physical health (0–10)	2.77	2.74	0.31 ( *p* ≤ 0.001)	0.23 (*p* ≤ 0.001)	0.22 (<0.001)
Perceived climate impact on mental health (yes/no)	0.64	0.48	0.30 (*p* ≤ 0.001)	0.25 (*p* < 0.001)	0.18 (<0.001)
Extent of perceived climate impact on mental health (0–10)	2.88	2.95	0.41 (*p* = < 0.001)	0.32 (*p* < 0.001)	0.26 (<0.001)

Note. Pearson correlation coefficients (r) are reported with *p*-values. All correlations were significant at *p* < 0.001.

## Data Availability

The datasets generated and analyzed during the current study are archived in hard-copy and electronic form at the Department of Neuroscience, Imaging and Clinical Sciences, “G. d’Annunzio” University of Chieti-Pescara. Because they contain information that could compromise participant confidentiality, the raw data are not publicly available but can be obtained from the corresponding author upon reasonable request and with permission of the Departmental Data Protection Officer.

## Supplementary Materials

The following supporting information can be downloaded at: https://www.mdpi.com/article/10.3390/ijerph22121768/s1, Table S1: Descriptive statistics and distributional properties of main study variables (N = 1051).
