# Supplementary material for: Eco-Anxiety and Mental Health: Correlates of Climate Change Distress
Source: Int J Environ Res Public Health. 2025 Nov 21;22(12):1768. doi: 10.3390/ijerph22121768 (PMC12732531; doi:10.3390/ijerph22121768)
Supplement: Supplementary file 1 [file ijerph-22-01768-s001.zip › ijerph-3903119-supplementary.pdf]

Table S1. Descriptive statistics and distributional properties of main study variables (N = 1051)

Variable	M	SD	Min	Max	Skewness (g_1)	Kurtosis (g_2)	Shapiro–Wilk W	p
**HEAS total (Eco-anxiety)**	7.22	7.14	0	39	1.20	1.33	0.89	< .001
**BSI total (Psychological distress)**	39.92	35.43	0	179	1.16	0.94	0.88	< .001
**PQ-16 total (Psychosis-risk indicators)**	9.14	9.16	0	48	1.31	1.49	0.90	< .001
**SF-36 total (Quality of life)**	95.44	13.37	0	124	–3.12	15.47	0.63	< .001
**Age (years)**	34.25	15.01	15	89	0.91	–0.42	0.86	< .001
**Gender (0 = male; 1 = female)**	0.69	0.46	0	1	–0.83	–1.31	0.58	< .001
**Education (ordinal)**	3.79	1.09	1	6	0.49	–0.54	0.88	< .001
**Has children (0 = no; 1 = yes)**	0.52	0.82	0	2	1.10	–0.60	0.61	< .001
**Chronic physical condition (0 = no; 1 = yes)**	0.16	0.37	0	1	1.86	1.46	0.44	< .001

Note. *M* = mean; *SD* = standard deviation. Skewness and kurtosis are unbiased sample estimates (Fisher's definitions). Shapiro–Wilk tests were significant for most variables due to the large sample size; interpretation was based on $|g_1| \leq 2$ and $|g_2| \leq 7$ as acceptable limits for normality.
